# Iron Dyshomeostasis and Ferroptosis: A New Alzheimer’s Disease Hypothesis?

**DOI:** 10.3389/fnagi.2022.830569

**Published:** 2022-03-22

**Authors:** Feixue Wang, Jiandong Wang, Ying Shen, Hao Li, Wolf-Dieter Rausch, Xiaobo Huang

**Affiliations:** ^1^Department of Traditional Chinese Medicine, Xuanwu Hospital, Capital Medical University, Beijing, China; ^2^Beijing Geriatric Institute of Integrated Traditional and Western Medicine, Beijing, China; ^3^Department of General Diseases, Wangjing Hospital, China Academy of Chinese Medical Sciences, Beijing, China; ^4^Department of Biomedical Sciences, Institute of Medical Biochemistry, University of Veterinary Medicine Vienna, Vienna, Austria

**Keywords:** Alzheimer’s disease, iron, ferroptosis, FPN1, GPX4, lipid peroxidation, iron chelator, hepcidin

## Abstract

Iron plays a crucial role in many physiological processes of the human body, but iron is continuously deposited in the brain as we age. Early studies found iron overload is directly proportional to cognitive decline in Alzheimer’s disease (AD). Amyloid precursor protein (APP) and tau protein, both of which are related to the AD pathogenesis, are associated with brain iron metabolism. A variety of iron metabolism-related proteins have been found to be abnormally expressed in the brains of AD patients and mouse models, resulting in iron deposition and promoting AD progression. Amyloid β (Aβ) and hyperphosphorylated tau, two pathological hallmarks of AD, can also promote iron deposition in the brain, forming a vicious cycle of AD development-iron deposition. Iron deposition and the subsequent ferroptosis has been found to be a potential mechanism underlying neuronal loss in many neurodegenerative diseases. Iron chelators, antioxidants and hepcidin were found useful for treating AD, which represents an important direction for AD treatment research and drug development in the future. The review explored the deep connection between iron dysregulation and AD pathogenesis, discussed the potential of new hypothesis related to iron dyshomeostasis and ferroptosis, and summarized the therapeutics capable of targeting iron, with the expectation to draw more attention of iron dysregulation and corresponding drug development.

## Introduction

Iron is the most abundant transition element on Earth (Coby et al., 2011) and one of the most important minerals in the body. It plays an indispensable role in many physiological and pathological processes of the body. Iron homeostasis is even more crucial in the brain to maintain its normal function. Iron dyshomeostasis within the brain can cause oxidative stress and inflammatory responses, leading to cell damage and finally neurological diseases. Ferroptosis, a programmed cell death process associated with iron dysregulation, has been supposed to be linked to neurological diseases, especially neurodegenerative diseases (Ren et al., 2020). Alzheimer’s disease (AD) is the most common type of dementia, and there is no effective clinical drug yet that can delay its progression, posing a serious burden on patients’ families and the whole society. β-amyloid (Aβ) plaque and tau tangles are the pathological hallmarks of AD, accompanied by neuronal loss and damage to brain tissue (Braak and Braak, 1991). Growing evidence have proved that Aβ triggers the chain events downstream in the pathological course of AD, including tau pathology. However, other processes such as neural inflammation (Leng and Edison, 2021), oxidative stress (Song et al., 2021), apoptosis (Sharma et al., 2021), autophagy (Zhang et al., 2021) and metal dysregulation (Wang et al., 2020; Aaseth et al., 2021) are also found in AD pathology. Some of these processes are even involved in the deposition of Aβ. Up to date, it is impossible to explain AD with a single pathological path. Currently, metal dyshomeostasis in AD has been extensively studied (Wang et al., 2020). Studies have found intracellular iron deposition even before the formation of senile plaques (Cheignon et al., 2018) and neurofibrillary tangles (NFT) (Wan et al., 2019) and ferroptosis is proposed to be one of key causes of neuronal loss in AD patients (Ashraf et al., 2020; Park et al., 2021). This review aimed to discuss the associations of AD pathogenesis with iron dyshomeostasis and ferroptosis, in order to highlight the hypothesis regarding iron-induced AD and the possibility of targeting iron and ferroptosis to treat AD.

## Iron Metabolism in Healthy and Alzheimer’s Disease Brain

About 48% of the iron in the body is bound to hemoglobin and is involved in oxygen transport in the body (Andreini et al., 2018). About 17% of the iron is found as the cofactor in proteins (Andreini et al., 2018) to carry out functions in several crucial biological processes such as the tricarboxylic acid cycle, oxidative phosphorylation, DNA synthesis and repair, and iron homeostasis (Sheftel et al., 2012; Andreini et al., 2018). In the brain, iron is involved in myelination, neurotransmitter synthesis, and antioxidant enzyme function, and its entry and exit are tightly regulated by a variety of molecules. Aging (Loeffler et al., 1995), inflammation (Yoshida et al., 1998; Urrutia et al., 2013), and oxidative stress, that disturb the functions of molecules involved in iron metabolism, present as the main contributors to iron dyshomeostasis.

### Iron Transport Across Blood-Brain Barrier

In the brain, transferrin receptor 1 (TfR1), responsible for the strict control of the level of iron transported into the brain (Pardridge et al., 1987; Connor et al., 2011), is expressed on the luminal side of the brain microvascular endothelial cells (BMECs) and the blood-cerebrospinal fluid barrier. After circulating iron forming a complex (holo-Tf) with transferrin (Tf), it binds to TfR1 on the surface of the BMECs, followed by entry into the BMECs via clathrin-mediated endocytosis (Pardridge et al., 1987; McCarthy and Kosman, 2012). Fe^3+^ detaches from Tf in the acidic environment of the endosome and is reduced to Fe^2+^ by six-transmembrane epithelial antigen of prostrate 3 (STEAP3) (Ohgami et al., 2005) or duodenal cytochrome b (DCYTB) (Tulpule et al., 2010), both of which are metalloreductases. It then enters the cytoplasm via divalent metal transporter 1 (DMT1) (De Domenico et al., 2008). Fe^2+^ in BMECs can then enter the brain by the secretion of ferroportin 1 (FPN1) followed by the oxidation by extracellular ceruloplasmin (Cp) or hephaestin (McCarthy and Kosman, 2013; Burkhart et al., 2016). There is also a hypothesis that iron could be transported to the brain interstitium through transcytosis in the form of holo-Tf based on the observation of lack of DMT1 in BMECs (Moos et al., 2007), which is still controversial. Not transferrin-bond iron (NTBI) can cross the Blood-Brain Barrier (BBB) via receptor-mediated transcytosis after binding to heavy-chain ferritin (H-ferritin; Hulet et al., 1999a,b) or lactoferrin (Lf) (Fillebeen et al., 1999; Fisher et al., 2007). It was also reported that Lf increased in the brains of aged individuals and those with AD (Kawamata et al., 1993), allowing large amounts of non-Tf-bound iron to enter the brain.

### Iron Transport and Storage Within the Brain

#### Neuronal Iron Metabolism

TfR1 is highly expressed on the surface of neurons (Giometto et al., 1990) and, similar to BMECs, iron enter neurons via clathrin-mediated endocytosis of holo-Tf/TfR1 and exit the endosomes in the form of reduced Fe^2+^ via DMT1 (Burdo et al., 2001). NTBI can also enter neurons in a DMT1-dependent manner independent of Tf. Cellular prion protein (PrPC) is abundantly expressed on the surface of neuronal membranes. It functions as a ferrireductase partner for DMT1, mediating Fe^2+^ uptake in the plasma membrane in the form of complex PrPc/DMT1 (Singh, 2014; Tripathi et al., 2015). PrPC knockout in mice can lead to iron deficiency in brain and uptake increase of holo-Tf (Singh et al., 2013). By comparing the brain tissues of juvenile, adult, and aged rats that had the pathological features of AD, Lu et al. (2017) found that DMT1 abnormally increased with age. They supposed that DMT1 may be one of the main reason why the iron concentration in the brain gradually increases with age (Lu et al., 2017).

Some Fe^2+^ undergoes normal metabolism in the cytoplasm of neurons, while some is stored in ferritin in the form of non-toxic Fe^3+^ (Nikseresht et al., 2019); when neurons are low in iron, ferritin can be degraded by lysosomes to release the stored iron to meet the physiological needs of the neurons (Mancias et al., 2014; Quiles Del Rey and Mancias, 2019). Ferritin is positively correlated with iron overload and is found deposited in senile plaques in the AD brain (Connor et al., 1992; Raha et al., 2022). It had been shown that there was an age-dependent increase in ferritin in the brain (Belaidi et al., 2018), probably a contributor to the iron overload in aged and AD brains. Autopsy studies of AD patients have revealed that mitochondrial ferritin is upregulated (Wang et al., 2011). Ferritin in the cerebrospinal fluid (CSF) of AD patients has been shown significantly increased, which is negatively correlated with cognitive decline and hippocampal atrophy in AD (Diouf et al., 2019). Additionally, iron can enter mitochondria to form iron sulfur cluster and participate in participate in the process of aerobic respiration (Peng et al., 2021).

Regarding the transport of excess iron out of neurons, FPN1 is the only known iron exporter (Ganz, 2005) to date. Both Cp and hephaestin (Heph) can oxidize Fe^2+^ and facilitate FPN1to export iron (Qian et al., 2007), so the FPN1/Cp and FPN1/Heph are the main iron efflux pathways (Dlouhy et al., 2019; Qian and Ke, 2019). Decrease of any of these three export proteins can induce iron retention and consequently the memory impairment (Jiang et al., 2015; Zheng et al., 2018; Bao et al., 2021). It was reported that FPN1 was downregulated in the brains of AD patients and triple-transgenic AD mouse models (Raha et al., 2013), thus excessive iron could not be excreted normally, initiating intracellular iron deposition. Since Cp is a crucial partner of FPN1 to oxidize Fe^2+^ before it is excreted by FPN1, the dysfunction of Cp serves as an upstream event of iron retention, which has been found in AD (Connor et al., 1993; Kristinsson et al., 2012; Zhao et al., 2018).

Noteworthy, both of amyloid precursor protein (APP) and tau, which are the substrates of the AD hallmarks in pathological condition, are crucial for neuronal iron efflux (McCarthy et al., 2014; Belaidi et al., 2018). APP is defined as a metalloprotein involved in iron homeostasis (Duce et al., 2010). With the assistance of soluble tau protein, APP is transported to the cell membrane (Lei et al., 2012) where it stabilizes FPN1 (Wong et al., 2014b) and facilitates the efflux of iron. APP or tau knockdown can lead to abnormal FPN1 function and the inability of neuronal iron to flow out normally, resulting in neuronal iron overload (Lei et al., 2012; Belaidi et al., 2018; Tsatsanis et al., 2020). APP with the pathogenic Italian mutation A673V is more prone to be cleaved by β-secretase to produce Aβ1–42, impeding its support of FPN1 and thus increasing iron retention (Tsatsanis et al., 2020). Because of the continuous cleavage of APP and hyperphosphorylation of tau in AD brain, the iron efflux was hindered in neurons (Tuo et al., 2017; Tsatsanis et al., 2020).

#### Glial Support for Neuronal Iron Metabolism

Glial cells help to maintain the iron availability at a safe level in neurons. Astrocytes and microglia respond during iron overload or deficiency in order to maintain neuronal iron homeostasis. As a buffer pool, astrocytes express abundant TfR1 and DMT1 (Pelizzoni et al., 2013; Zarruk et al., 2015), which facilitates taking up both of holo-Tf and NTBI from the abluminal side of BMECs and the brain interstitium, precisely regulating the iron concentration in neurons (Tulpule et al., 2010; Hohnholt and Dringen, 2013; Knutson, 2019). Microglia also express TfR1 and reduce iron toxicity by promoting the influx of excess iron (for storage in ferritin) via the TfR1/DMT1 pathway (Qian and Ke, 2019). Microglia and astrocytes are capable of releasing ferritin carrying Fe^3+^ to supplement the iron deficiency or to support oligodendrocytes for myelination or remyelination (Cheli et al., 2021). Iron is essential for myelination in oligodendrocytes, which are the most iron-rich cell type in the brain. TfR1 is absent in oligodendrocytes (Zhang et al., 2006), while H-ferritin is the main source of iron for oligodendrocyte by interaction with T-cell immunoglobulin mucin domain 2 (TIM2) (Todorich et al., 2009, 2011). Noteworthy, when iron is overloaded, oligodendrocytes provide an antioxidant defense for neurons by secreting H-ferritin, scavenging extracellular extra iron (Mukherjee et al., 2020).

### Regulators of Iron Homeostasis Proteins in the Brain

#### Transcription Factors and Iron Regulatory Element/Iron Regulatory Protein Interaction

At the transcriptional level, many of the intracellular iron homeostasis-related genes described above can be regulated by hypoxia-inducible factorα (HIFα). Most of the mRNAs related to the intracerebral iron metabolism have a hairpin-like structure known as iron regulatory element (IRE) (Camaschella, 2019). The iron regulatory protein 1/2 (IRP1/2) is the iron sensor, which can bind to the IRE, post-transcriptionally regulating those iron homeostasis-related proteins. In a low iron or hypoxic state in cells, HIFα is activated and upregulates IRP. IRP then binds to the IRE at the 3′-untranslated region (UTR) end of TfR1 and DMT1 mRNAs, to promote their translation, and to the IRE at the 5′-UTR end of ferritin, FPN1, and APP mRNAs, to inhibit their translation (Rogers et al., 2002; Anderson and Frazer, 2017). As a result, iron intake is increased, stored iron is released, and iron efflux is reduced. However, under iron overload, HIFα is degraded, and the IRP-IRE interactions lead to iron storage in neurons and increased iron efflux (Meyron-Holtz et al., 2004). Nuclear factor E2-related factor 2 (Nrf2) is an important regulator of intracellular oxidative stress and is also involved in the regulation of iron metabolism-related proteins. Nrf2 is able to upregulate FPN1 (Chen et al., 2020), while Nrf2 knockout reduces FPN1 levels in BMECs and prevents iron entry into the brain (Han et al., 2021). Decrease of Nrf2 could be one of the means for defense against iron overload in the brain (Han et al., 2021). Nevertheless, most of the target genes of Nrf2 have antioxidative property (Hayes and Dinkova-Kostova, 2014), thereby endowing it as a potent inhibitor of lipid oxidation and ferroptosis (Sun et al., 2016). This will be discussed in the part of “Ferroptosis and AD.”

#### Hepcidin

Hepcidin is a polypeptide that plays a major role in regulating peripheral iron homeostasis, and it has also been shown to be expressed in cortical neurons, intracerebral BMECs, and glial cells (Zechel et al., 2006; Raha et al., 2013; Raha-Chowdhury et al., 2015), playing an important role in the regulation of iron homeostasis in the brain. It binds to FPN1 in the cell membrane of BMECs, and then the hepcidin/FPN1 complex is internalized and degraded (Drakesmith et al., 2015) so that iron cannot cross the BBB into the brain. From this perspective, hepcidin is beneficial in preventing iron overload in neuron and maintaining iron homeostasis in the brain (Du et al., 2015; Vela, 2018). Studies have found hepcidin decreased in AD affected neurons (Raha et al., 2013), while exogenous hepcidin supplement normalized the levels of iron transport proteins, thereby reducing iron level in the brain (Du et al., 2015). The AD symptoms and Aβ burden can be ameliorated by astrocyte hepcidin (Xu et al., 2020; Zhang et al., 2020). Therefore, it is partially accepted that hepcidin is protective as an iron homeostasis regulator in the brain. However, it was also reported that hepcidin mRNA and protein increased in the brain with aging (Wang et al., 2010); it can be up-regulated by inflammation (Urrutia et al., 2013), especially by interleukin-6, with the result of FPN1 decrease and iron retention in AD brains (Chaudhary et al., 2021), exerting the detrimental impact on the brain. Up to date, studies on hepcidin in brain iron regulation remain controversial. How hepcidin allowing the normal iron efflux from neurons while causing FPN1 degradation remains an important issue to be studied.

#### Apolipoprotein E

Apolipoprotein E (apoE) is a protein responsible for the cholesterol transport to neurons, and also regulates Aβ transport and deposition. The allele ε4 of *APOE* (*APOE4)* is the strongest genetic risk factor for AD. CSF ferritin level is positively correlated with apoE4 expression, and it can be enhanced in *APOE4* carriers (Ayton et al., 2015, 2017a). In old-aged individuals, cortical iron burden was found more severe by quantitative susceptibility mapping in *APOE4* carriers (Kagerer et al., 2020). Therefore, brain iron deposition caused by *APOE4* may be one of the reasons why *APOE4* allele predisposes individuals to AD (Wood, 2015). Interestingly, increased extracellular iron levels can upregulate *APOE4* expression in the neurons, but reduce the secretion of it, thereby reducing its ability to clear extracellular Aβ and aggravating Aβ deposition (Xu et al., 2016). From recent studies, iron could be the link between *APOE4* and AD.

## Impact of Iron Overload on Alzheimer’s Disease Pathology

Alzheimer’s disease is characterized by senile plaque and NFT pathologically. Senile plaque derives from the accumulation of insoluble Aβ, which is cleaved from APP by β-secretase. Hyperphosphorylation of tau is proposed to be the downstream event of Aβ formation. The pathological mechanism of AD is too complex to explain it merely with Aβ and tau accumulation. Scientists have found that many pathological processes, for instance, neural inflammation (Leng and Edison, 2021), oxidative stress (Song et al., 2021) and metal dysregulation (Wang et al., 2020; Aaseth et al., 2021), involved in Aβ deposition and tau hyperphosphorylation, driving the insidious progression before AD diagnosis. Currently, the involvement of iron in the early pathology of AD has been well accepted since the discovery of the link between dysregulation of brain iron homeostasis and AD pathogenesis in 1953 (Goodman, 1953).

In the preclinical stage of AD, there is significant abnormal iron elevation in cortical, hippocampal, and cerebellar neurons (Ding et al., 2009; Smith et al., 2010), while much severer in the cortex and hippocampus, the main brain areas affected by AD (Smith et al., 1997; Belaidi and Bush, 2016). The iron overload in the brain is corresponding to the severity of AD lesions and the rate of cognitive decline (van Duijn et al., 2017; Ayton et al., 2020). It is also proposed that hippocampal iron deposition could be the predictor of the rate of cognitive decline caused by Aβ (Ayton et al., 2017b). Iron overload drives a series of events, including glial activation, formation of Aβ plaque and tau tangles, and even neuronal loss (Singh et al., 2014; Gleason and Bush, 2021), pushing the progress of the disease and accelerating cognitive decline.

### Iron Interaction With Aβ Plaques and Neurofibrillary Tangles

Iron accumulation was demonstrated to accelerate senile plaque deposition and the production of neurofibrillary tangles (Becerril-Ortega et al., 2014; Kim et al., 2018). Autopsy evidence and magnetic resonance imaging analysis provide evidence that there are a large amount of iron deposition not only in and around senile plaques (James et al., 2017), but also in the sites of cortical tau accumulation (Spotorno et al., 2020), indicating the potential crosstalk of iron with both of senile plaques and neurofibrillary tangles.

Perturbations in iron homeostasis is one of key players in Aβ deposition. High intracellular iron concentration enhances the interaction of IRP/IRE, inducing APP upregulation. Furthermore, the enzymes that cleave APP named α- and β-secretase are tightly balanced and modulated by furin (Silvestri and Camaschella, 2008; Guillemot et al., 2013). More β-secretase is activated when α- secretase is suppressed by furin impairment in the condition of excessive iron (Silvestri and Camaschella, 2008). Upregulated APP is cleaved by more β-secretase to Aβ40/42, accelerating the Aβ deposition. Meanwhile, APP can no longer assist FPN1, resulting in impaired iron efflux and aggravated iron deposition (Ward et al., 2014; Greenough, 2016). Some researchers have even proposed that Aβ is non-toxic in the absence of redox metals and that aggregation of Aβ requires the involvement of metals (Li et al., 2013; Belaidi and Bush, 2016). Soluble Aβ binds to Fe^3+^ when extracellular iron increases, so as to remove excess iron, but it is difficult to dissociate them after they interact; Aβ can promote the reduction of Fe^3+^ to Fe^2+^, and the reactive oxygen species (ROS) released during this process allows Aβ to be deposited more easily and rapidly, forming more senile plaques (Ha et al., 2007). The interactions of iron with APP and Aβ greatly increase the formation rate and degree of senile plaques (Rottkamp et al., 2001; Ha et al., 2007). Therefore, some researchers believe that iron deposition should be included in the “Aβ cascade hypothesis” of AD (Peters et al., 2015; Gleason and Bush, 2021).

Iron can also interact with tau. Reduced soluble tau in the brain of AD patients increased brain iron deposition by suppressing FPN1 activity (Lei et al., 2012). On the contrary, a diet high in iron can lead to cognitive decline in mice, increased abnormal tau phosphorylation in neurons, and abnormal expression of insulin pathway-related proteins. Insulin supplementation can reduce iron-induced phosphorylation of tau (Wan et al., 2019), indicating that iron deposition may lead to tau hyperphosphorylation by interfering insulin signaling. *In vivo* research has found that iron can be involved in tau hyperphosphorylation by activating the cyclin-dependent kinase 5 (CDK5)/P25 complex and glycogen synthase kinase-3β (GSK-3β) (Guo et al., 2013a). Excessive intracellular Fe^2+^-induced production of oxygen free radicals can also promote tau hyperphosphorylation by activating the extracellular signal-regulated kinase 1/2 (Erk1/2) or mitogen-activated protein kinase (MAPK) signaling pathways (Chan and Shea, 2006; Munoz et al., 2006).

### Iron Overload and Glial Activation

Glial activation and neuroinflammation has been demonstrated to be a prominent characteristic of AD pathology (Newcombe et al., 2018; Leng and Edison, 2021). Microglial are highly reactive cells responding to increased iron levels in the brain. When iron level increases in brain, microglia become activated (Meng et al., 2017), with soma volume increased and process length decreased (Rathnasamy et al., 2013; Donley et al., 2021). Iron may activate microglia through proinflammatory cytokines mediated by the nuclear factor-κB (NF-κB) (Meng et al., 2017). After activated, they express more ferritin to scavenge the extracellular iron (Streit et al., 2021), resulting in intracellular iron retention (Kenkhuis et al., 2021), increased TNFα expression (Holland et al., 2018), and finally infiltrated with Aβ-plaques (Peters et al., 2018; Kenkhuis et al., 2021). Activated microglia also secret Lf, which can interact with APP, promoting the Aβ formation (Tsatsanis et al., 2021). Conversely, formation of Aβ induces more IL-1β expression in microglia in the environment of elevated iron, exacerbating the proinflammatory effects (Nnah et al., 2020).

Astrocytes are highly resistant to metal-induced toxicity within the brain (Kress et al., 2002) as the critical cell type in maintaining a balanced extracellular environment and supporting the normal functioning of neurons (Abbott et al., 2006). In the environment of high iron, astrocytes respond with a significant increase in glutathione, catalase and manganese superoxide dismutase levels to resist the oxidative stress (Iwata-Ichikawa et al., 1999; Shih et al., 2003). They show less impairment by iron than neurons and oligodendrocytes (Kress et al., 2002). But later, the astrocytes were found activated with increased glial fibrillary acidic protein (GFAP) (Kress et al., 2002). Activated astrocytes release inflammatory mediators and induce oxidative stress, which facilitate the formation of Aβ and tau tangles and hinder Aβ clearance (Cai et al., 2017).

### Iron Overload Induces Oxidative Stress and Neuronal Loss

Iron toxicity is largely based on Fenton chemistry. As a redox-active transition metal, iron is a key player during the process of oxidative stress. Elevated iron promotes the production of ROS, which further depletes the cellular antioxidant GSH and promotes lipid peroxidation (Maher, 2018), finally triggering ferroptosis and neuronal loss. This will be discussed in the next part.

## Ferroptosis and Alzheimer’s Disease

### Ferroptosis in Brain

Ferroptosis is a programmed cell death process discovered by Dixon et al. (2012) that is distinct from other modalities of cell death such as apoptosis and necrosis. Ferroptosis is characterized by iron overload and triggered by iron-driven pile-up of the lipid peroxides to a lethal level (Dixon et al., 2012). The morphological changes of cells undergoing ferroptosis include cell swelling, reduced or absent mitochondrial cristae, decreased mitochondrial membrane density, nuclear chromatin condensation, and finally mitochondrial and cellular membrane rupture (Xie et al., 2016). It can be halted by iron chelators, antioxidants, and ferroptosis blockers.

With age, iron is gradually deposited in the body and iron deposition in the brain is more pronounced than in other organs. In addition, brain contains the highest level of polyunsaturated fatty acids (PUFAs), which are the preferential substrate for lipid peroxidation in the process of ferroptosis. So it is not surprising that brain is more susceptible to undergo ferroptosis. So far, ferroptosis has been found in neurodegenerative diseases (Masaldan et al., 2019), cerebrovascular diseases (Guan et al., 2019; Abdul et al., 2021), and other central nervous system diseases (Weiland et al., 2019).

It remains elusive of the precise role of iron even though it is indispensable in the execution of ferroptosis. Fe^2+^ itself has oxidation ability; it can produce a large amount of ROS via the Fenton reaction when it accumulates in excess (Wong et al., 2014a). This directly induces peroxidation of PUFA and leads to the lethal accumulation of the hydride PUFA-OOH (Angeli et al., 2017). Additionally, iron accumulation can activate iron-dependent lipoxygenases (LOXs) (Yang et al., 2016), thus excessively catalyzing the enzymatic oxidation of PUFA. Neuronal ferroptosis can be caused by both mechanisms.

Glutathione peroxidase 4 (GPX4) is the primary enzyme that negatively regulate ferroptosis. It physiologically reduces PUFA-OOH to non-toxic PUFA-OH (Ashraf et al., 2020), thus playing a detoxifying role and preventing ferroptosis. The intracellular level of glutathione (GSH; comprising cysteine, glycine, and glutamate), which is used by GPX4, is affected by the intracellular cysteine level. When the glutamate/cystine antiporter (xCT) encoded by *SLC7A11* is inhibited under the condition of inflammation, aging, and other factors, the level of intracellular cysteine is reduced, resulting in insufficient GSH for the biosynthesis of GPX4 (Dixon et al., 2014). Excessive PUFA-OOH cannot be detoxified and accumulates to a lethal level, triggering ferroptosis (Weiland et al., 2019). Therefore, the dysfunction of xCT or GPX4 represents an upstream factor in neural ferroptosis (Ashraf et al., 2020). Restoration the functions of GPX4, GSH, or xCT has been demonstrated to be one of the strategies to inhibit ferroptosis (Seibt et al., 2019).

If ferritin, the major iron storage protein in cells, binds to its cargo receptor, nuclear receptor coactivator 4 (NCOA4) under the condition of inflammation, it is transported to lysosomes for autophagic degradation (Gao et al., 2016; Quiles Del Rey and Mancias, 2019), followed by the entry of iron into the cytoplasm. This is an important trigger for ferroptosis in cells. It has been found that multiple autophagy genes were associated with ferroptosis, while blocking or knocking down these genes reduced ferroptosis (Park and Chung, 2019). Thus, ferroptosis is considered to be an autophagic process.

### “Ferroptosis Hypothesis” of Alzheimer’s Disease

After the discovery of ferroptosis in 2012, research on ferroptosis continued, and the involvement of ferroptosis in neurodegenerative diseases has now been widely accepted. Ferroptosis may be a potential mechanism of neuronal loss in many neurodegenerative diseases (Hou et al., 2019; Ayton et al., 2021; Bao et al., 2021; Tian et al., 2021). Ayton et al. (2021) proposed the “ferroptosis hypothesis” in AD, which could be independent of the “Aβ hypothesis” and “tau protein hypothesis,” based on large cohort studies. Thus, iron and ferroptosis are supposed to be the promising therapeutic targets for treating neurodegenerative diseases.

Raha et al. (2013) found reduced hippocampal FPN1 expression in AD patients and AD transgenic mice, but the association with ferroptosis was not examined. Bao et al. (2021) found that FPN1 was decreased in the brains of APP/presenilin-1(PS1) mice and AD patients; moreover, genetic *FPN1* deletion in the neurons of neocortex and hippocampus in NEX-Cre mice led to AD-like atrophy in the hippocampus and cognitive impairment. The characteristic neuronal morphology of ferroptosis was observed; the biomarkers of ferroptosis named acyl-CoA synthetase family member 2 (ACSF2) and iron response element binding protein 2 (IREB2) were both upregulated; GPX4 was downregulated in *FPN1* deletion mice as well as in APP/PS1 mice (Bao et al., 2021). FPN1 restoration or ferroptosis inhibitors named liproxstatin-1 and ferrostatin-1 improved memory impairment in both types of the mice (Bao et al., 2021). This not only indicated that abnormal expression of FPN1 solely is capable of inducing AD, but also implicated the role of ferroptosis in the pathogenesis of AD.

The mRNA *GPX4* and its protein level have been both found to be aberrantly expressed in the brains of AD patients and mice (Yoo et al., 2010; da Rocha et al., 2018). In glial cells, a mild hypoxic state is capable of decreasing the level of GSH, which is used for the biosynthesis of GPX4 (Makarov et al., 2006). In AD mouse models, GSH expression is reduced in the cortex and is positively correlated with cognitive decline (Karelson et al., 2001). Some researchers have proposed that GSH levels in the frontal lobe and hippocampus may serve as a biomarker to predict AD and mild cognitive impairment (Mandal et al., 2015; Ayton et al., 2020). The xCT activity determines GSH availability and, subsequently GPX4 activity in the brain (Ashraf et al., 2020). Furthermore, studies have found that most of the proteins that involved in ferroptosis can be regulated by Nrf2 (Habib et al., 2015; Lane et al., 2021). The target genes include FPN1, GSH as well as *SLC7A11* that encode xCT. Nrf2 level in the brain decreases with age, and even worse in the brains of AD (Osama et al., 2020), representing a possible upstream that leads to ferroptosis (Habib et al., 2015). GPX4 expression was reported to be reduced in the brains of both AD mouse models and AD patients (Ansari and Scheff, 2010; Yoo et al., 2010). *GPX4*-knockout mice were proved to have significant hippocampal neuronal loss and cognitive impairment (Yoo et al., 2012; Hambright et al., 2017); the diet deficient in vitamin E, an antioxidant with anti-ferroptosis activity, deteriorated hippocampal neurodegeneration and behavior dysfunction; ferroptosis inhibitors liproxstatin 1 improved the cognitive function and ameliorated the neurodegeneration in these mice (Hambright et al., 2017).

**FIGURE 1:**
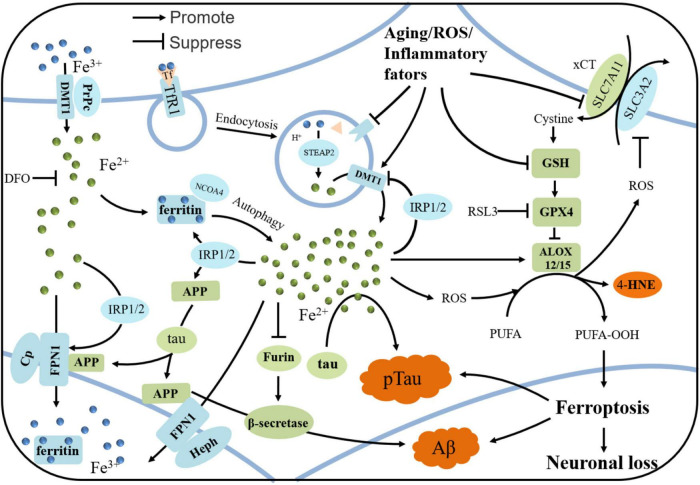
The iron regulation in neuron and the mechanism of ferroptosis in AD. Iron is uptaken by neurons via holo-Tf/TfR1 complex or DMT1/PrPC-dependent manners. PrPC reduce Fe^3+^ to Fe^2+^ and DMT1 transport Fe^2+^ into neurons. Tf carrying Fe^3+^ forms a complex with TfR1, and enters neurons via clathrin-mediated endocytosis. Fe^3+^ detaches from Tf and then is reduced by STEAP3. Fe^2+^ is pumped to cytoplasm by DMT1, and is stored in ferritin in the form of Fe^3+^ when overload. In some conditions, ferritin undergoes autophagy by binding with NCOA4, releasing iron and subsequently leading to lethal iron levels and ferroptosis. Elevated irons could be excreted by FPN1/Cp or FPN1/Heph, with the assistance of APP, which is transported to stabilize FPN1 by soluble tau protein. Aging, inflammation, and oxidative stress could dysregulate the iron transport proteins and cause iron retention. When overload, iron could upregulates the expressions of ferritin, FPN1, and APP by IRP-IRE interactions, while suppresses the normal function of furin, leading to the upregulation of β-secretase and thus accelerating Aβ deposition. When xCT or GSH decreases in the neurons under some states, the decreased GPX4 cannot exert the function of anti-lipid peroxidation. After Fenton reaction or ALOX-catalyzed process, PUFA-OOH can accumulate to a lethal level to trigger ferroptosis, which could be responsible for the tau hyperphosphorylation, Aβ formation and neuronal loss.

In addition to findings in animal models, autopsy findings revealed that GPX4 was downregulated and arachidonate 12/15-lipoxygenase (ALOX15) was upregulated, and the enhanced lipid peroxidation was observed, evidenced by elevated 4-hydroxynonenal (4-HNE) in the brains of AD patients (Yoo et al., 2010). 4-HNE has the potential to modify the proteins that involved in the antioxidant and energy metabolism, promoting Aβ deposition and fibril formation (Siegel et al., 2007). Thus, it is evident that ferroptosis plays a key role in AD, causing neuronal loss and cognitive decline (the roles of iron dyshomeostasis and ferroptosis are shown in Figure 1). Therefore, regulating brain iron metabolism and reducing neuronal ferroptosis may be a promising treatment for AD.

## Targeting Iron Dyshomeostasis and Ferroptosis to Treat Alzheimer’s Disease

### Iron Chelators

#### Deferoxamine and Deferiprone

Deferoxamine (DFO; also known as desferrioxamin) is an iron chelator that is commonly used in clinical practice. It may be an effective treatment for AD, and animal experiments and clinical trials have been conducted on DFO. Crapper et al. (1991) conducted a single-blind trial of intramuscular DFO in 48 AD patients for 24 months, and they found that DFO reduced the rate of clinical progression of AD by up to 50% compared to the control group. However, DFO also caused weight and appetite loss (Crapper et al., 1991).

Animal experiments involving the systemic administration of DFO to treat AD are rare, and whether DFO can cross the BBB has not yet been conclusively established. However, some studies have found that DFO crosses the BBB less efficiently than other iron chelators (Ward et al., 1995; Shachar et al., 2004), so nasal administration may be a better choice. Nasal administration of DFO has been found to reduce Aβ deposition and cognitive dysfunction in AD models (Fine et al., 2012, 2015; Guo et al., 2013b). However, nasal administration can cause gastrointestinal deposition of DFO, and there is inadequate penetration through the nasal mucosa thus low bioavailability (Lochhead and Thorne, 2012). Nasal administration of DFO nanoparticles may be a safer and effective way to treat AD (Rassu et al., 2015). This is currently being studied and may be used for treating AD in the near future.

Deferiprone (DFP) is a chelator that could be absorbed orally and cross BBB (Hider and Hoffbrand, 2018). In a randomized controlled trial, DFP improved neurological scores and iron-related neurological symptoms (Abbruzzese et al., 2011; Klopstock et al., 2019). DFP is currently being investigated in a randomized, double-blinded, placebo controlled, and multi-central phase II trial involving patients with mild cognitive impairment, prodromal AD or mild AD, who should have sedimentary Aβ confirmed by PET scan (NCT03234686). Giving the safety and lower systemic toxicities, DFP is a viable strategy to treat AD against iron dyshomeostasis and ferroptosis.

#### Clioquinol and Its Derivatives

Clioquinol and its derivatives, which chelate iron, zinc, and copper, have also been shown to improve cognitive ability, reduce Aβ deposition, and/or promote Aβ degradation in AD animal models (Grossi et al., 2009; Crouch et al., 2011). This may be related to their capture of iron from Aβ and prevention of Aβ aggregation (Opazo et al., 2006). It has also been found that clioquinol downregulates β- and γ-secretase and APP expression in the brain (Wang et al., 2012). *In vitro* experiment demonstrated that clioquinol was able to degrade oligomerized tau and reduce tau tangles (Lin et al., 2021). The effect of clioquinol in AD was also validated in a randomized controlled trial involving patients with moderate-to-severe AD, as oral clioquinol compared to placebo led to slower cognitive decline and reduced CSF Aβ42 (Ritchie et al., 2003). There is a derivative of clioquinol that have been studied extensively named PBT2 [5,7-dichloro-2-((dimethylamino)methyl) 8-quinolinol]. PBT2 has the effect to promote Aβ degradation (Crouch et al., 2011). A phase IIa, double-blind, randomized, placebo-controlled trial showed satisfactory safety and tolerability of PBT2 in AD, with the significant reduction in CSF Aβ42 concentration and improvement in cognition (Lannfelt et al., 2008; Faux et al., 2010). Because of the potential of clioquinol in treating AD, some derivatives or hybrids were synthesized to treat AD, and were proved effective both *in vitro* and *in vivo* (Perez et al., 2019). Yang et al. (2020) tested a series of novel flurbiprofen-clioquinol hybrids synthesized for Alzheimer’s disease therapy *in vitro*, and found they could prevent the accumulation of Aβ with antioxidant and anti-neuroinflammatory activity.

Clioquinol and its derivatives or hybrids were demonstrated to treat AD in the above studies ascribed to their chelation properties for zinc and copper. Other studies indicated clioquinol was also capable of preventing the loss of substantia nigra cells in the Parkinsonian transgenic mouse model because of its ability to chelate iron (Billings et al., 2016). Further studies on clioquinol to chelate iron in the treatment of AD need to be conducted in the future. Giving the roles of the three metals in AD, clioquinol chelating three of them should performed better in the treatment of AD. Because of the comparably less side-effects, clioquinol seems more promising than DFO for treating AD.

### Antioxidants

#### Vitamin E

Antioxidants have been widely used to treat AD, and many of them can inhibit lipid peroxidation and thus ferroptosis. Vitamin E has been found to reduce lipid peroxidation in the brains of *GPX4*-knockout mice, mitigate the ferroptosic morphology of neurons, and improve the cognitive function of the mice. However, vitamin E didn’t show any benefit in patients with AD or mild cognitive impairment in a randomized clinical trial (Farina et al., 2017). Moreover, another clinical trial found that vitamin E treatment accelerated cognitive decline compared to placebo (Lloret et al., 2009). Therefore, the use of vitamin E in AD remains questionable and more clinical trials are needed to ascertain its effect.

#### Alpha-Lipoic Acid

Alpha-lipoic acid (α-LA) is an antioxidant with benefits for AD. In a clinical study of 129 patients with possible AD, α-LA slowed cognitive decline and reduced cognitive impairment (Fava et al., 2013). α-LA relieved tau hyperphosphorylation in P301S tau transgenic mice, and it also blocked tau-induced iron overload, lipid peroxidation, and inflammation (Zhang et al., 2018), thus exerting the effect to ameliorate ferroptosis in the brain. As no serious side effects of α-LA have been observed so far, α-LA may be useful for developing treatments for AD.

#### Selenium

Selenium (Se) is present in a variety of proteins in the body, such as GPX4, and it has antioxidant activity. Research have found that decreased Se in the brain of AD patients may be associated with AD progression (Cardoso et al., 2017; Varikasuvu et al., 2019). Primary neuron culture indicated that Se might reduce Aβ production by reducing 4-HNE-induced β-secretase transcription, thereby preventing Aβ-mediated toxicity (Gwon et al., 2010). Se -containing compounds may inhibit ferroptosis by upregulating GPX4. A clinical trial of 40 AD patients found that, on average, oral sodium selenate effectively supplemented brain Se levels without significant side effects, and among those who responded (i.e., had improved brain selenium levels), the Mini-Mental State Examination (MMSE) score did not significantly deteriorate over time (Cardoso et al., 2019). However, Kryscio et al. (2017) studied the effects of Se and vitamin E on AD progression in male patients in the PREADViSE trial and found that each of them had deleterious effects. In summary, the current knowledge provides no valid evidence for a beneficial role of Se in the treatment of AD, and clinical trials are still needed to provide definitive answers.

#### Ferrostatin-1

Ferrostatin-1 (Fer-1), a radical scavenger, is a common ferroptosis inhibitor much more efficiently than phenolic antioxidants (Miotto et al., 2020). It has been demonstrated to inhibit oxidative lipid damage and cell death in diverse disease models, including Huntington’s disease, periventricular leukomalacia, kidney dysfunction, intracerebral hemorrhage, and cardiomyopathy (Skouta et al., 2014; Li et al., 2017; Fang et al., 2019). Fer-1 was able to alleviate angiotensin II -induced inflammation and ferroptosis in astrocytes by suppressing the ROS levels and downregulating Nrf2 and GPX4 (Li et al., 2021). In the treatment of AD, Fer-1 was evidenced to ameliorate neuronal death and memory impairment both *in vitro* and *in vivo* (Bao et al., 2021). After traumatic brain injury, the mice administrated Fer-1 was shown significantly reduced iron deposition and neuronal degeneration in the brains as well as improved long-term motor and cognitive function (Xie et al., 2019). Although *in vivo* and *in vitro* studies have proved the pronounced effect of Fer-1 in ameliorating oxidative stress and preventing ferroptosis, there is still no clinical trials as yet. Its exact efficacy in AD needs to be further excavated as well as its side-effects.

### Hepcidin

As hepcidin can reduce the transport of iron across the BBB and prevent iron overload in the brain, some researchers have proposed that it can treat AD, which has been confirmed in cell-based and animal experiments. In cultured microvascular endothelial cells, hepcidin significantly inhibited FPN1, and consequently low expression of TfR1 and DMT1, as well as reduced iron uptake and iron release in neurons (Du et al., 2015). In APP/PS1 mice, overexpressing hepcidin in astrocytes reduced neuronal iron level in cortex and hippocampus, where the formation of Aβ plaque were also alleviated; the mice cognitive function was elevated (Xu et al., 2020). Consistently, another study found that treatment with recombinant adenovirus carrying the hepcidin gene reduced iron retention and oxidative stress in the brain (Gong et al., 2016). These studies demonstrated the excellent potential of hepcidin in AD treatment, but hepcidin is currently only being investigated in preclinical studies. How to deliver hepcidin to brain and its side-effects yet need to be further studied and solid evidenced.

## Conclusion and Future Prospects

As the most common type of dementia, AD has become a major challenge for society and health systems because the globe population is rapidly aging. Most clinical studies of treatments based on the mainstream hypotheses regarding AD pathogenesis have not been highly fruitful. The gradual understanding of the roles of iron dyshomeostasis and ferroptosis in the pathogenesis of AD may offer a promising direction to establish a new hypothesis, which will be of great significance and worthy to be studied deeper. In the future, the upstream and downstream roles of iron in AD pathogenesis are yet to be elucidated, and iron quantification methods will definitely be improved with technological advances to assist the scientists better understand the iron dyshomeostasis in different brain regions. Even though a various of molecules have been found to have the potential to alleviate iron dyshomeostasis and prevent ferroptosis, finding a way to allow these potentially useful drugs, such as iron chelators and antioxidants, to effectively cross the BBB into the brain in order to mediate their effects while avoiding systemic side effects will be the greatest challenge for future pharmacological research on AD treatment based on the potential “ferroptosis hypothesis.”

## Author Contributions

XH: conceptualization. FW, JW, and YS: methodology and investigation. JW, HL, W-DR, and XH: validation. FW: writing – original draft preparation. FW, HL, W-DR, and XH: writing – review and editing. YS and XH: supervision. YS: project administration. FW, YS, and XH: funding acquisition. All authors: contributed to the article and approved the submitted version.

## Conflict of Interest

The authors declare that the research was conducted in the absence of any commercial or financial relationships that could be construed as a potential conflict of interest.

## Publisher’s Note

All claims expressed in this article are solely those of the authors and do not necessarily represent those of their affiliated organizations, or those of the publisher, the editors and the reviewers. Any product that may be evaluated in this article, or claim that may be made by its manufacturer, is not guaranteed or endorsed by the publisher.
